# Validation of the spanish version of the multiple sclerosis international quality of life (musiqol) questionnaire

**DOI:** 10.1186/1471-2377-11-127

**Published:** 2011-10-18

**Authors:** Oscar Fernández, Victoria Fernández, Karine Baumstarck-Barrau, Luis Muñoz, Maria del Mar Gonzalez Alvarez, José Carlos Arrabal, Antonio León, Ana Alonso, Jose Carlos López-Madrona, Rafael Bustamante, Gloria Luque, Miguel Guerrero, Elisabetta Verdun di Cantogno, Pascal Auquier

**Affiliations:** 1 Institute of Clinical Neurosciences. Service of Neurology. Hospital Regional Universitario Carlos Haya, Avda. Carlos Haya s/n, 29010 Málaga, Spain; 2 Institute of Clinical Neurosciences. Service of Neurophysiology. Hospital Regional Universitario Carlos Haya, Avda. Carlos Haya s/n, 29010 Málaga, Spain; 3 Department of Public Health, EA3279 Research Unit, University Hospital, Boulevard Jean Moulin, 13385 Marseille, France; 4 Global Medical Affairs Neurology, Merck Serono S.A., 9 Chemin des Mines, 1202 Geneva, Switzerland

## Abstract

**Background:**

The Multiple Sclerosis International Quality Of Life (MusiQoL) questionnaire, a 31-item, multidimensional, self-administrated questionnaire that is available in 14 languages including Spanish, has been validated using a large international sample. We investigated the validity and reliability of the Spanish version of MusiQoL in Spain.

**Methods:**

Consecutive patients with different types and severities of multiple sclerosis (MS) were recruited from 22 centres across Spain. All patients completed the MusiQoL questionnaire, the 36-Item Short Form (SF-36) health survey, and a symptoms checklist at baseline and 21 days later. External validity, internal consistency, reliability and reproducibility were tested.

**Results:**

A total of 224 Spanish patients were evaluated. Dimensions of MusiQoL generally demonstrated a high internal consistency (Cronbach's alpha: 0.70-0.92 for all but two MusiQoL domain scores). External validity testing revealed that the MusiQoL index score correlated significantly with all SF-36 dimension scores (Pearson's correlation: 0.46-0.76), reproducibility was satisfactory (intraclass correlation coefficient: 0.60-0.91), acceptability was high, and the time taken to complete the 31-item questionnaire was reasonable (mean [standard deviation]: 9.8 [11.8] minutes).

**Conclusions:**

The Spanish version of the MusiQoL questionnaire appears to be a valid and reliable instrument for measuring quality of life in patients with MS in Spain and constitutes a useful instrument to measure health-related quality of life in the clinical setting.

## Background

Health-related quality-of-life (HRQoL) measurements have become an important instrument in population health assessment, treatment evaluation and care management [1-3]. HRQoL indicators depend on the completion of a well-validated questionnaire that assesses health status in individual patients as perceived by themselves through the physical, mental, social and behavioural components of well-being and function [4]. Thus, one major challenge in elaborating the content of the HRQoL dimensions to be measured is to ensure that patients' perceptions are accurately taken into account [5]. Specific HRQoL questionnaires that are available for MS in Spain are FAMS (Functional Assessment of Multiple Sclerosis) [6] and MSQOL-54 (Multiple Sclerosis Quality of Life 54) [7]. However, both of these are based on generic QoL instruments.

The Multiple Sclerosis International Quality of Life (MusiQoL) questionnaire, a specific, self-administered, multidimensional questionnaire, was co-developed and initially validated in 15 countries including Spain [8]. The initial item pool of the Functional Assessment of Multiple Sclerosis included items from the general version of the Functional Assessment of Cancer Therapy quality of life instrument. The other items were generated by patients, providers, and literature review. The MSQOL-54 has been adapted from the SF36 measure by the addition of five unchanged SF36 scales, three "altered" SF36 scales (one item added to each scale), and five new scales incorporated 15 additional items. Modifying existing measures by adding MS-specific items was not found to be as useful. The content of the QoL questionnaires is now well-recognized as more appropriate when it is derived from the patients' point of view/patients' interviews. MusiQoL questionnaire specifically reflects the point of view of patients with MS on the impact of the disease on their daily life and is anchored in an explicit conceptual approach as recommended by McKenna [5]. We investigated the validity and reliability of the Spanish version of MusiQoL in Spain.

## Methods

### Patients

The patients enrolled in the validation study consisted of inpatients and outpatients who were followed in an international multicentre study coordinated by an international steering committee (15 senior neurologists, two experts in HRQoL, one expert in health economics and two external advisors). The inclusion criteria were a diagnosis of MS according to Poser [9] or McDonald [10] criteria as the main diagnosis for more than 6 months, age older than 18 years, informed consent to participate in the study and Spanish as the native language. The main exclusion criteria included a main diagnosis other than MS, dementia, current severe relapses, inability to complete the questionnaire independently and withdrawal of consent. Patients were enrolled between January 2004 and February 2005 from 22 centres distributed throughout Spain.

### Study design

Patients were evaluated at inclusion and re-tested a mean (standard deviation) of 21 (7) days later. The self-administered survey materials that were completed by the patients included the MusiQoL questionnaire, one checklist of 14 symptoms reported by patients suffering from MS (e.g. lack of sensation in touch, lack of sensation in position, fatigue, visual problems, urinary incontinence) and the 36-Item Short Form (SF-36) health survey [11].

The SF-36 is the most widely used generic QoL scale in MS, consisting of 36 items that describe eight dimensions: Physical Functioning (PF), Social Functioning (SF), Role-Physical Problems (RPP), Role-Emotional Problems (REP), Mental Health (MH), Vitality (VIT), Bodily Pain (BP), and General Health (GH). Each dimension is scored from 0-100; the higher the score, the better the perceived state of health.

MusiQoL comprises 31 items that describe nine dimensions (Additional file 1 and Additional file 2). Each dimension is named according to its constitutive items, as follows: activities of daily living (ADL, 8 items), psychological well-being (PWB, 4 items), symptoms (SPT, 3 items), relationships with friends (RFr, 4 items), relationships with family (RFa, 3 items), relationship with the healthcare system (RHCS, 3 items), sentimental and sexual life (SSL, 2 items), coping (COP, 2 items) and rejection (REJ, 2 items). Each item was answered using a six-point Likert scale, where 1 = 'never/not at all', 2 = 'rarely/a little', 3 = 'sometimes/somewhat', 4 = 'often/a lot', 5 = 'always/very much', 6 = 'not applicable'. The negatively worded item scores were reversed so that higher scores indicated a higher level of HRQoL. For each patient, the score of each dimension was obtained by computing the mean of the item scores of the dimension. If fewer than half of the items were missing, the mean of the non-missing items was substituted for the missing items. All dimension scores were linearly transformed to a 0-100 scale. A global index score was computed as the mean of the dimension scores.

In addition, an experienced local neurologist collected sociodemographic data, clinical history (related or unrelated to MS), and information on the type of treatment for MS. The neurologist also rated the following: Poser classification (or McDonald classification), the Expanded Disability Status Scale (EDSS) [12], the Ambulation Index (AI) for MS [13], the Folstein Mini-Mental State Examination (MMSE) [14] and the Clinical Global Impression (CGI) of severity scale (mild, moderate, severe) [15]. A detailed physical examination was conducted at baseline and any abnormalities in the organ systems inspected were noted.

At re-testing, patients completed the same questionnaire and one additional question assessing changes in health status. Neurologists collected data on current care and treatment of MS, rated the scores for the EDSS and CGI, and assessed patient health status compared with that reported at the initial inclusion stage (i.e. worsened, remaining stable, improved).

### Statistical analyses

The linguistic transcultural equivalence was ensured by the codevelopment process, including interview and selection of items conducted in Spain.

The multidimensional structure (construct validity) of the Spanish version of the MusiQoL questionnaire was checked using multitrait/multi-item analysis program (MAP) [16] analyses. Internal structural validity was assessed using item-dimension correlations: item internal consistency (IIC) was assessed by correlating each item with its scale (correlation of 0.4 for supporting item internal consistency [IIC]) and item discriminant validity (IDV) was assessed by determining the extent to which items correlates with the dimension they are hypothesized to represent than with the other ones. Floor and ceiling effects were assessed by the homogeneous repartition of the response distribution. For each dimension, internal consistency reliability was assessed by Cronbach's alpha coefficient. A Cronbach's alpha coefficient of at least 0.7 was expected for each scale [17]. The unidimensionality of each scale was assessed using Rasch analyses: item goodness-of-fit statistics (INFIT) and coefficient of Loevinger (H). INFIT ranging between 0.7 and 1.2 and H of at least 0.40 ensure that all the items of the scale tend to measure the same concept.

To explore external validity, relations between: i) dimensions of MusiQoL and the SF-36, and ii) dimensions of MusiQoL and the 14-symptom scale assessing miscellaneous domains, were examined using Pearson's correlation coefficients (*r)*. The underlying assumption was that MusiQoL dimension scores would be more correlated with the scores of similar dimensions from the other instruments than with the scores of dissimilar dimensions [8]. The discriminant validity was determined by assessing the associations between the MusiQoL dimension scores and sociodemographic and clinical features. For qualitative variables, mean dimension scores of the MusiQoL were compared across patient groups that were expected to differ (e.g. MS forms, CGI, gender, family status, educational level, employment status, housing) using one-way analysis of variance. Quantitative variables (e.g. MMSE, AI, EDSS score, age) were analysed using Pearson's correlation coefficients.

Reproducibility was tested through test-re-test reliability using intraclass correlation coefficients (ICC) between the two successive assessments in patients with stable clinical disease. Stable clinical disease was defined using both the physician clinical global impression and the EDSS.

Acceptability was assessed by calculating the average time of completion of the MusiQoL questionnaire and the percentage of missing data per dimension.

Data analyses were performed using SPSS 11.0, MAP-R, and WINSTEP software.

## Results

### Patient characteristics

The study sample included 224 Spanish patients with MS (Table 1). The mean (standard deviation [SD]) age was 39 (10) years (min = 20/max = 63). More than 75% of patients had clinically definite MS according to Poser criteria. Almost 80% were patients with the relapsing-remitting form of MS, and there were no patients with clinically isolated syndrome. Mean EDSS was 2.9 (SD: 2.0; range 0-8) with a mean disease duration of 11 years (range: 1-36).

**Table 1:** Baseline characteristics of the study population (n = 224)

Variable		n (%)
Age, mean (SD), years (n = 224)		39 (10)
Sex (n = 223)	Female	157 (70.4)
	Male	66 (29.6)
Poser classification (n = 223)	Clinically defined MS	172 (77.1)
	Laboratory defined MS	49 (22.0)
	Clinically probable MS	2 (0.1)
Clinical form of MS (n = 222)	Relapsing-remitting	176 (79.3)
	Secondary progressive	40 (18.0)
	Primary progressive	6 (2.7)
Disease duration, mean (SD), range, years		10.6 (7.2) 1-36
EDSS score, mean (SD), range		2.9 (1.9) 0-8

EDSS, Expanded Disability Status Scale; MS, multiple sclerosis; SD, standard deviation.

### Construct Validity and Internal Structural Validity

The correlation of each item was higher with the MusiQoL dimension of which it was part than with any other dimension, except for the items of the coping dimension. Floor and ceiling effects can be considered as satisfactory (ceiling effects of RFa and RHCS dimensions were high, 33.0 and 41.1 respectively; Table 2). Dimensions of the MusiQoL showed satisfactory internal consistency (Cronbach's alpha: 0.70-0.92), except for the dimensions of RFa (Cronbach's alpha: 0.67) and RHCS (Cronbach's alpha: 0.53; Table 2).

**Table 2:** MusiQoL dimension scale characteristics

Dimension (number of items)	Mean (SD)	MV (%)	Cronbach's alpha	**IIC**, range	**IDV**, range	Floor effect, %	Ceiling effect, %	H	INFIT
Activities of Daily Living (8)	57.04 (25.96)	1.8	0.92	0.67-0.80	-0.02 to 0.51	0.9	4.5	0.65	0.87-1.19
Psychological Well-Being (4)	52.95 (23.80)	0.4	0.88	0.70-0.79	-0.01 to 0.60	2.2	4.0	0.68	0.80-1.12
Relationships with Friends (3)	68.61 (22.51)	6.3	0.80	0.59-0.73	-0.02 to 0.33	1.3	13.4	0.61	0.91-1.12
Symptoms (4)	66.45 (22.64)	0.9	0.70	0.43-0.59	-0.02 to 0.51	0.0	11.2	0.37	0.79-1.20
Relationships with Family (3)	82.92 (17.82)	1.3	0.67	0.47-0.56	0.01-0.43	0.0	33.0	0.44	0.98-1.02
Relationship with Healthcare System (3)	86.79 (16.02)	0.4	0.53	0.25-0.40	-0.02 to 0.23	0.0	41.1	0.30	0.82-1.15
Sentimental and Sexual Life (2)	73.14 (26.52)	16.1	0.76	0.65	0.04-0.38	3.1	25.0	0.68	0.91-1.10
Coping (2)	59.31 (28.02)	5.4	0.75	0.60	0.13-0.65	3.1	15.2	0.65	0.96-1.01
Rejection (2)	83.11 (21.62)	7.1	0.75	0.66	0.10-0.43	0.0	1.3	0.56	0.94-1.04
Index	70.32 (13.71)								

H Loevinger coefficient; IDV, item discriminant validity; IIC, item internal consistency; INFIT item goodness-of-fit (Rasch statistics); MV, percentage of missing values; SD, standard deviation.

The overall scalability was satisfactory except for 2 dimensions: the SPT dimension showed an INFIT statistic and a Loevinger coefficient H outside the acceptable ranges, the RHCS dimension provided a coefficient H inferior to 0.40.

### External validity

The concepts covered by the MusiQoL and the SF-36 are not largely overlapping. MusiQoL dimension scores were only moderately correlated with scores of the SF-36, except for expected correlation of ADL with physical functioning and vitality; and PWB and COP with MH (p < 0.05; Table 3). The situation was similar when comparing the MusiQoL dimensions with the self-reported 14-symptom scale. ADL on MusiQoL was, at the most, moderately correlated with all components of the 14-symptom scale (p < 0.05), except for 'inability to swallow' (Table 3).

**Table 3:** Pearson's correlations between MusiQoL and SF-36, and the 14 self-reported symptom scales

	MusiQoL dimensions									
**SF-36 dimensions**	**ADL**	**PWB**	**RFr**	**SPT**	**RFa**	**RHCS**	**SSL**	**COP**	**REJ**	**Index**
Physical functioning	0.729**	0.203**	0.087	0.311**	0.178**	0.217**	0.195**	0.282**	0.248**	0.496**
Social functioning	0.515**	0.347**	0.212**	0.441**	0.178**	0.198**	0.275**	0.373**	0.222**	0.541**
Role - physical	0.603**	0.409**	0.078	0.514**	0.181**	0.177**	0.118	0.315**	0.300**	0.551**
Role - emotional	0.443**	0.443**	0.099	0.527**	0.115	0.126	0.164*	0.489**	0.274**	0.559**
Mental health	0.383**	0.665**	0.241**	0.504**	0.310**	0.145*	0.389**	0.654**	0.465**	0.760**
Vitality	0.688**	0.577**	0.157*	0.550**	0.258**	0.214**	0.354**	0.532**	0.364**	0.699**
Bodily pain	0.431**	0.392**	0.064	0.471**	0.149*	0.180**	0.157*	0.283**	0.219**	0.463**
General health	0.542**	0.347**	0.128	0.360**	0.116	0.200**	0.265**	0.450**	0.247**	0.521**

**14-symptom scales**	**ADL**	**PWB**	**RFr**	**SPT**	**RFa**	**RHCS**	**SSL**	**COP**	**REJ**	**Index**

Lack of sensation in touch	-0.295**	-0.057	0.070	-0.112	0.068	0.126	-0.160	0.062	-0.019	-0.100
Lack of sensation in position	-0.332**	-0.087	0.109	-0.192*	0.030	0.063	-0.134	-0.066	-0.030	-0.173
Involuntary body movements	-0.433**	-0.083	0.040	-0.133	-0.073	-0.018	0.001	-0.213*	-0.105	-0.249*
Vibration in legs or arms	-0.462**	-0.185*	-0.060	-0.274**	-0.083	-0.092	-0.083	-0.204*	-0.167	-0.348**
Weakness in limbs	-0.575**	-0.106	-0.047	-0.214**	-0.031	-0.048	0.000	-0.147	-0.149	-0.242**
Tingling in limbs	-0.329**	-0.132	0.025	-0.246**	-0.040	-0.071	-0.010	-0.067	-0.127	-0.272**
Inability to swallow	-0.054	-0.155	-0.011	-0.056	-0.232	-0.080	-0.202	-0.279*	-0.052	-0.269*
Involuntary eye movements	-0.215*	-0.064	0.020	-0.205	0.102	0.082	-0.046	-0.102	0.032	-0.151
Visual problems	-0.311**	-0.185*	0.051	-0.423**	0.062	-0.062	-0.138	-0.087	-0.124	-0.321**
Problems concentrating	-0.425**	-0.364**	-0.114	-0.587**	-0.050	-0.112	-0.134	-0.323**	-0.236**	-0.522**
Difficulty concentrating	-0.326**	-0.224*	-0.235*	-0.395**	-0.048	-0.060	0.001	-0.234*	-0.388**	-0.422**
Fatigue	-0.553**	-0.222**	-0.075	-0.336**	-0.004	-0.034	-0.077	-0.303**	-0.184*	-0.373**
Urinary incontinence	-0.404**	-0.118	-0.074	-0.220*	-0.078	-0.019	-0.212*	-0.058	-0.050	-0.239*
Bowel incontinence	-0.274*	-0.190	-0.020	-0.291**	-0.058	-0.113	-0.088	-0.046	-0.114	-0.182

Bold values indicate significant correlation.

*p < 0.05; **p < 0.01.

ADL, activities of daily living; COP, coping; PWB, psychological well-being; REJ, rejection; RFa, relationships with family; RFr, relationships with friends; RHCS, relationship with healthcare system; SPT, symptoms; SSL, sentimental and sexual life.

### Discriminant validity

The discriminant validity of MusiQoL was assessed by clinical and sociodemographic characteristics (Tables 4 and 5). There were significant differences based on MS classification in the dimensions of ADL, COP and REJ and the index score (p < 0.05). Patients with secondary progressive MS generally had lower scores (34.5-74.7) than did patients with relapsing-remitting MS (RRMS) (62.4-85.4) or primary progressive MS (31.0-81.3). Differences were also observed when comparing dimension scores by severity rated on the CGI; patients with mild (56.1-88.1) or moderate (47.8-87.4) disease scored higher than those with severe disease (32.6-80.0) for all the domains including the index.

**Table 4:** MusiQoL dimension scores, by clinical characteristics and medical history

		MusiQoL dimension									
Variable		ADL	PWB	RFr	SPT	RFa	RHCS	SSL	COP	REJ	Index
***MS classification***											
**Relapsing-remitting (n = 176)**	**Mean**	63.5	54.3	69.3	67.7	83.5	87.3	73.6	62.4	85.4	72.0
	**SD**	24.0	23.4	22.5	22.2	17.5	16.5	25.8	26.2	20.0	13.0
**Primary progressive (n = 6)**	**Mean**	31.0	50.0	76.4	65.6	81.9	80.6	72.5	57.5	81.3	65.0
	**SD**	18.5	22.0	13.4	28.2	25.0	17.2	37.9	31.4	25.9	9.2
**Secondary progressive (n = 40)**	**Mean**	34.5	49.1	64.9	62.9	80.1	85.0	70.5	46.9	74.7	63.3
	**SD**	18.9	24.7	23.7	23.2	18.7	13.7	29.7	31.8	25.6	15.9
	**p-value**	< 0.001	0.435	0.402	0.475	0.569	0.464	0.854	0.009	0.021	0.011

***Clinical global impression***											
**Mild (n = 118)**	**Mean**	68.7	56.1	68.2	70.8	83.0	88.1	74.0	63.5	87.0	73.2
	**SD**	23.9	24.0	23.8	22.1	17.8	14.3	26.0	26.4	19.8	13.2
**Moderate (n = 70)**	**Mean**	47.8	50.2	70.3	61.1	85.4	87.4	75.0	61.2	80.0	69.0
	**SD**	20.5	22.4	21.9	20.7	17.5	17.5	25.5	28.2	22.4	12.5
**Severe (n = 24)**	**Mean**	32.6	49.3	69.4	62.8	73.6	80.0	63.3	40.9	74.4	59.9
	**SD**	17.6	23.6	18.5	27.4	18.6	17.7	27.7	28.1	26.0	15.4
	**p-value**	**< 0.001**	0.166	0.843	**0.012**	**0.023**	0.074	0.284	**0.002**	**0.015**	**0.003**

***MMSE (n = 221)***	**r**	0.29	0.15	0.05	0.23	0.04	0.12	0.09	0.15	0.25	0.26
	**p-value**	**< 0.001**	0.029	0.488	**0.001**	0.575	0.085	0.204	**0.028**	**< 0.001**	**0.001**

***Ambulation Index (n = 220)***	**r**	-**0.62**	-0.12	0.00	-0.19	-0.16	-0.17	-0.07	-0.26	-0.20	-0.34
	**p-value**	**< 0.001**	0.075	0.985	**0.006**	**0.022**	**0.011**	0.378	**< 0.001**	**0.005**	**< 0.001**

***EDSS (n = 221)***	**r**	**-0.63**	**-0.14**	0.02	-0.25	-0.04	-0.18	-0.06	**-0.29**	**-0.26**	**-0.35**
	**p-value**	**< 0.001**	**0.045**	0.789	**< 0.001**	0.511	**0.008**	0.399	**0.001**	**< 0.001**	**< 0.001**

Bold values: p < 0.05.

ADL, activities of daily living; ANOVA, analysis of variance; CIS, clinically isolated syndrome; COP, coping; EDSS, Expanded Disability Status Scale; MMSE, Mini Mental Score Examination; PWB, psychological well-being; r, correlation coefficient; REJ, rejection; RFa, relationships with family; RFr, relationships with friends; RHCS, relationship with healthcare system; SPT, symptoms; SSL, sentimental and sexual life.

**Table 5:** MusiQoL dimension scores, by sociodemographic characteristics

Variable		ADL	PWB	RFr	SPT	RFa	RHCS	SSL	COP	REJ	Index
***Sex***											
**Female (n = 157)**	**Mean**	58.4	51.0	69.8	66.0	82.7	86.8	74.6	58.8	83.8	70.5
	**SD**	25.4	24.0	21.0	23.0	18.4	16.0	26.2	27.8	22.1	13.6
**Male (n = 66)**	**Mean**	54.5	58.4	66.3	68.1	83.3	86.6	70.2	61.3	81.7	70.5
	**SD**	26.8	21.9	25.6	21.5	16.8	16.2	27.3	27.9	20.8	13.8
	**p-value**	0.316	**0.033**	0.311	0.541	0.821	0.915	0.292	0.552	0.538	0.998
***Family status***											
**Couple (n = 145)**	**Mean**	52.8	50.5	68.7	64.7	83.6	86.5	75.6	60.0	82.8	69.5
	**SD**	26.1	22.7	21.2	22.7	18.1	16.3	23.9	28.0	22.1	13.8
**Single (n = 33)**	**Mean**	60.9	58.1	70.7	71.7	77.0	90.4	60.7	60.2	85.6	70.6
	**SD**	21.4	20.8	19.2	14.9	19.9	13.0	30.4	26.8	16.9	12.0
**With relatives or family (n = 45)**	**Mean**	69.0	58.1	67.5	69.3	85.2	84.9	70.8	57.8	82.4	74.8
	**SD**	24.4	27.3	28.3	26.1	14.6	17.1	32.9	28.4	23.8	13.6
	**p-value**	**0.001**	0.072	0.837	0.190	0.104	0.309	**0.0048**	0.899	0.776	0.214

***Education level***											
**Primary (n = 69)**	**Mean**	49.8	47.4	68.5	62.0	83.0	88.3	75.2	57.1	82.3	67.8
	**SD**	27.0	20.9	21.9	23.1	18.4	15.7	25.5	29.6	24.7	13.1
**Secondary (n = 82)**	**Mean**	57.1	48.9	64.8	63.5	80.1	83.2	68.6	56.3	80.3	68.1
	**SD**	23.9	24.6	22.5	20.6	20.2	19.1	27.2	26.2	23.4	14.0
**Tertiary (n = 69)**	**Mean**	64.1	63.2	73.4	74.6	85.5	89.4	76.1	66.3	87.3	75.6
	**SD**	25.2	21.6	22.5	22.6	14.0	11.2	27.1	26.5	14.7	12.2
	**p-value**	**0.005**	**< 0.001**	0.074	**0.001**	0.181	**0.040**	0.223	0.062	0.155	**0.004**

***Employment status***											
**Student (n = 17)**	**Mean**	72.9	55.3	74.0	76.8	88.7	90.7	77.1	55.9	90.6	75.8
	**SD**	21.5	19.3	20.8	21.7	12.1	11.7	24.3	28.0	9.7	10.2
**Worker (n = 104)**	**Mean**	64.0	57.0	70.2	71.2	83.5	86.6	75.1	65.2	87.0	73.9
	**SD**	25.4	22.2	23.4	21.2	17.1	15.3	24.4	27.7	19.4	12.5
**Unemployed (n = 102)**	**Mean**	47.7	48.9	66.4	60.3	81.3	86.2	70.4	54.6	78.2	66.2
	**SD**	23.6	25.1	21.8	22.4	19.3	17.3	29.2	27.0	24.1	14.0
	**p-value**	**< 0.001**	**0.047**	0.319	**< 0.001**	0.260	0.562	0.442	**0.025**	**0.007**	**0.001**

***Housing***											
**Own home (n = 168)**	**Mean**	53.3	51.9	69.6	64.8	82.5	86.2	74.8	59.8	83.3	69.6
	**SD**	25.3	22.6	21.0	22.5	18.5	16.5	24.9	27.8	21.2	13.7
**With relatives (n = 54)**	**Mean**	70.1	57.8	66.2	72.5	84.3	88.6	66.8	59.4	83.7	74.3
	**SD**	23.3	25.8	26.7	21.9	15.8	14.8	32.3	28.0	22.6	12.6
	**p-value**	**< 0.001**	0.109	0.345	**0.031**	0.526	0.336	0.097	0.935	0.914	0.075

***Age***	**r**	**-0.36**	-0.16	0.09	**-0.21**	-0.05	-0.03	-0.07	-0.01	-0.03	-0.32
	**p-value**	**< 0.001**	**0.019**	0.220	**0.002**	0.453	0.621	0.333	0.902	0.674	**0.006**

Bold values: p < 0.05.

ADL, activities of daily living; ANOVA, analysis of variance; COP, coping; DS, p < 0.05; NS, not significant; PWB, psychological well-being; r, correlation coefficient; REJ, rejection; RF, relationships with family; RFr, relationships with friends; RHCS, relationship with healthcare system; SPT, symptoms; SSL, sentimental and sexual life

A significant difference was shown in SPT scores in patients with cognitive problems (not including the exclusion criterion of dementia). The time from first symptoms did not correlate with any of the MusiQoL domains. EDSS was only moderately correlated with ADL dimension (p > 0.01) (Table 4). No significant differences were found according to family history of MS. Patients actually treated and patients treated for the symptoms of relapse did not show significant differences in HRQoL (data not shown).

Women were found to have recorded lower scores than men for PWB. Differences were observed when comparing ADL and SSL scores by family status, with patients living with relatives/family scoring higher for ADL, and couples scoring the highest for SSL and those living with relatives/family (p < 0.05). Patients with a tertiary education had higher scores in the dimensions of ADL, PWB, SPT and RHCS and the index score than did those with a primary or secondary education. Higher scores were also found among students and workers than those who were unemployed in the domains of ADL, PWB, SPT, COP and REJ and the index score. For the ADL and SPT domains, patients living with relatives had significantly higher scores than those living alone (Table 5).

### Reproducibility

The number of patients defined as stable between the 2 assessments was 172 according to the physician CGI definition and 190 according to the EDSS. The reproducibility of the MusiQoL domains scores and index score were satisfactory irrespective of the definition of stability, with intraclass correlation coefficients ranging from 0.60-0.91 (Table 6).

**Table 6:** Reproducibility of MusiQoL scores in patients with stable physician CGI (n = 172) and EDSS scores (n = 190)

	Physician CGI (n = 172)*	EDSS (n = 190)**
Activities of Daily Living	0.89	0.90
Psychological Well-Being	0.81	0.90
Relationships with Friends	0.73	0.73
Symptoms	0.83	0.84
Relationships with Family	0.60	0.76
Relationship with Healthcare System	0.69	0.69
Sentimental and Sexual Life	0.78	0.79
Coping	0.80	0.79
Rejection	0.86	0.84
Index	0.91	0.91

CGI, Clinical Global Impression; EDSS, Expanded Disability Status Scale.

### Acceptability

Acceptability was high. Mean (SD) time for completion of the MusiQoL items was 9.8 (11.8) minutes in the patient group as a whole. Mean (SD) time for completion was 9.7 (12.7) minutes in patients with RRMS, 11.6 (7.7) minutes in patients with primary progressive MS and 9.8 (7.4) minutes in patients with secondary progressive MS. The proportion of missing values per dimension was considered to be acceptable, ranging from 0.4 to 7.1% with the exception of the SSL dimension, which had 16.1% of values missing.

## Discussion

The MusiQoL study has been a major effort to develop a multidimensional specific HRQoL instrument for patients with MS. It has been developed simultaneously in 1992 patients from 15 countries and in 14 languages [8], using an standardized methodology [18]. It takes into account patients' concerns about QoL, extracting items such as sentimental and sexual life, which patients have described as important for them, and that are not included in other previous instruments [19,20].

Some HRQoL instruments have already been validated in patients with MS in Spain [6,7]. The Spanish version of FAMS was validated in a study that assessed 625 patients with MS recruited in an outpatient clinic setting from 58 hospitals in Spain. Of these, 74% were patients with RRMS, with a mean age of 37 years, a mean disease duration of 8.8 years and a mean EDSS of 3 points at baseline. The scale showed very good subscale homogeneity comparable to that of the original English version. The Spanish version was not tested for external validity with other HRQoL scales [6].

A transcultural adaptation of the MSQOL-54 has also been validated in a Spanish version. Ten interviews were carried out with 5 men and 5 women with MS, aged 21-54 years, with different education levels and EDSS scores ranging from 1.0 to 8.0. The translated version shows that the Spanish pre-test version is comprehensible and its administration feasible in patients with MS. The psychometric properties must be evaluated in the next phase of the project [7].

The Patient-Reported Indices for Multiple Sclerosis (PRIMUS) is a recently developed scale that comprises three scales for assessing symptoms, activity limitations and QoL in MS. It was translated using a dual-panel process and validated in eight languages, including Spanish. For the Spanish validation, 87 patients were tested, with a mean age of 43 years and 9 years of disease duration at baseline. Forty-seven percent of these patients had RRMS. No data about EDSS at the moment of the study are provided. Most of the tests showed good unidimensionality and internal consistency, reproducibility and construct validity in each translated language, including Spanish [21].

Our sample of Spanish patients with MS has similar clinical characteristics (80% RRMS, disease duration of 10.6 years and mean EDSS of 2.9) to the previously described MS samples [22], so they are perfectly comparable.

This study confirms the process of validation (external validity, internal consistency, reliability and reproducibility) of the Spanish version of the MusiQoL. Our results for validation are satisfactory and similar to those of the international patient sample [8]. This indicates a major strength of the MusiQoL regarding the simultaneous process of validation in different countries.

In the present study, the SF-36 was used for external validation. The SF-36 is a generic questionnaire that has previously been adequately validated in Spanish patients with other health conditions [23-30] and has been used occasionally in patients with MS [31-33]. MusiQoL dimension scores were, in our study, at most moderately correlated with the scores of SF-36, indicating that the two scales do not overlap.

The largest study using the SF-36 for the evaluation of QoL in Spanish patients with MS included 705 patients, 78% of whom had RRMS, with a mean age of 40.5 years and a median EDSS score of 2.5. These characteristics are similar to those of the patients included in our study and the scores for the eight SF-36 dimensions are also similar. The previous study reported lower physical QoL in patients with higher EDSS scores, but there was no apparent differences in the SF-36 dimensions when stratified by EDSS [31].

We found significant correlations between ADL scores and clinical indices such as EDSS and Ambulation Index, as other authors found [34,35], and the clinical global impression was significantly different in patients with mild or moderate disease, who scored higher than those with severe disease in every MusiQoL domain. These results are similar to the results found in the Spanish FAMS validation study where higher EDSS scores predicted worse scores on all aspect of QoL [8]. PWB score was higher for the male compared to the females. This is a usual result, reported by other authors [36]. These findings emphasize the usefulness of HRQoL instruments, specifically MusiQoL, and indicate the utility of the ADL dimension in reporting the clinical status of patients with MS from their own point of view [37].

The present study had several limitations. The population differs to that in the original study by Simeoni et al [8] as there were no patients with clinically isolated syndrome and only 6 patients with primary progressive MS, preventing the validity of the questionnaire from being evaluated in these groups. Further work could be conducted prospectively in a sample with representative proportions of patients in terms of clinical subtypes of MS. The missing values for most MusiQoL dimensions were considered to be acceptable (range: 0.4-7.1%), except for the SSL dimension, which had 16.1% missing values. This highlights that SSL may be an underreported deficit in patients with MS. The external validity was explored by studying relationships between dimensions of MusiQoL and dimensions of SF36, because the SF36 was the single questionnaire available in the needed versions for the initial international validation study; it could be completed by studying correlations of Musiqol and other widespread Spanish MS-specific instruments.

Although MusiQoL could be used with advantage in other Spanish-speaking countries, given its demonstrated acceptable cross-cultural validity [8], small adaptations and further validations would be necessary to ensure reliable application within each of those settings. Even with all these cautions, we point out its potential use as an outcome measure in clinical settings, particularly in multinational clinical trials.

## Conclusions

The Spanish version of the MusiQoL instrument has been shown to be a valid and reliable instrument for measuring HRQoL in patients with MS. Acceptability was high and time for completion short, and thus MusiQoL constitutes a useful instrument to measure HRQoL in the clinical setting in Spain.

## List of abbreviations

ADL: Activities of Daily Living; AI: Ambulation Index; BP: Bodily Pain; CGI: Clinical Global Impression; COP: Coping; EDSS: Expanded Disability Status Scale; FAMS: Functional Assessment of Multiple Sclerosis; GH: General Health; HRQoL: Health-Related Quality-of-Life; ICC: Intraclass Correlation Coefficients; IDV: Item Discriminant Validity; IIC: Item Internal Consistency; INFIT: Item goodness-of-fit; MAP: Multitrait/multi-item Analysis Program; MH: Mental Health; MMSE: Mini-Mental State Examination; MS: Multiple Sclerosis; MSQOL-54: Multiple Sclerosis Quality of Life-54 [questionnaire]; MusiQoL: Multiple Sclerosis International Quality Of Life [questionnaire]; PF: Physical Functioning; PRIMUS: Patient-Reported Indices for Multiple Sclerosis. PWB: Psychological Well-Being; QoL: quality-of-life; REJ: Rejection; REP: Role-Emotional Problems; RFa: Relationships with Family; RFr: Relationships with Friends; RHCS: Relationship with the HealthCare System; RPP: Role-Physical Problems; SD: Standard Deviation; SF: Social Functioning; SF-36: 36-Item Short Form [questionnaire]; SPT: Symptoms; SSL: Sentimental and Sexual Life; VIT: Vitality.

## Competing interests

**OF **has received honoraria for serving as a consultant in advisory boards, or chair or speaker in meetings; and for participation in clinical trials and other research projects promoted by Biogen-Idec, Bayer-Schering, Merck Serono, Teva and Novartis. VF, KBB, LM, MdMGA, JCA, AL, JCLM, RB, GL, MG and PA have no competing interests to declare. **EVdC **is a salaried employee of Merck Serono S.A. - Geneva, Switzerland.

## Authors' contributions

OF participated in study design and implementation in Spain, data collection and interpretation, and the writing of this manuscript. VF participated in the writing and interpretation of this manuscript. KBB and PA were instrumental to the study design, data analysis and interpretation of the results. EVdC participated in trial design and data collection, and interpretation of study findings. LM, MdMGA, JCA, AL, JCL-M, RB, GL, MG and the MusiQoL study group of Spain assisted with data collection. All authors had access to the study data and read and approved the final manuscript.

## Pre-publication history

The pre-publication history for this paper can be accessed here:

http://www.biomedcentral.com/1471-2377/11/127/prepub

## Supplementary Material

Additional file 1 **Table s1a: List of the 31 MusiQoL items (English version)**. Complete list of the 31 MusiQoL items used in the English version Click here for file

Additional file 2 **Table s1b**. List of the 31 MusiQoL items (Spanish version). Complete list of the 31 MusiQoL items used in the Spanish version Click here for file
