# Supplementary material for: Validation of the spanish version of the multiple sclerosis international quality of life (musiqol) questionnaire
Source: BMC Neurol. 2011 Oct 18;11:127. doi: 10.1186/1471-2377-11-127 (PMC3206836; doi:10.1186/1471-2377-11-127)
Supplement: Additional file 1 — Table s1a: List of the 31 MusiQoL items (English version). Complete list of the 31 MusiQoL items used in the English version [file 1471-2377-11-127-S1.DOC]

**Table 1a.** **List of the 31 MusiQoL items (English version).**

For each question, check the response that is closest to your feelings. Due to your MS, during the past 4 weeks, have you …

1. Had difficulty walking or moving outside?

2. Had difficulty with outdoor activities: i.e. shopping, going out to a movie?

3. Had difficulty walking or moving around at home?

4. Been troubled by your balance or walking problems?

5. Had difficulty with leisure activities at home: i.e. do-it-yourself, gardening?

6. Had difficulty with your occupational activities: i.e. integration, interruption, limitation?

7. Been quickly tired?

8. Been short of energy?

9. Felt anxious?

10. Felt depressed or gloomy?

11. Felt like crying?

12. Felt nervous or irritated by a few things or situations?

13. Been troubled by loss of memory?

14. Had difficulty concentrating: i.e. when reading, watching a film, following a discussion?

15. Been troubled by your vision: worsened or unpleasant?

16. Experienced unpleasant feelings: i.e. hot, cold?

17. Talked with your friends?

18. Felt understood by your friends?

19. Felt encouraged by your friends?

20. Talked with your spouse/partner or your family?

21. Felt understood by your spouse/partner or your family?

22. Felt encouraged by your spouse/partner or your family?

23. Felt satisfied with your love life?

24. Felt satisfied with your sex life?

25. Felt that your situation is unfair?

26. Felt bitter?

27. Been upset by the stares of other people?

28. Been embarrassed when in public?

29. Been satisfied with the information on your disease or the treatment given by the doctors, nurses, psychologists taking care of your MS?

30. Felt understood by the doctors, nurses, psychologists taking care of your MS?

31. Been satisfied with your treatments?
