# Supplementary material for: Validation of the spanish version of the multiple sclerosis international quality of life (musiqol) questionnaire
Source: BMC Neurol. 2011 Oct 18;11:127. doi: 10.1186/1471-2377-11-127 (PMC3206836; doi:10.1186/1471-2377-11-127)
Supplement: Additional file 2 — Table s1b. List of the 31 MusiQoL items (Spanish version). Complete list of the 31 MusiQoL items used in the Spanish version [file 1471-2377-11-127-S2.DOC]

**Table 1b. List of the 31 MusiQoL items (Spanish version).**

### *Para cada pregunta, marque la respuesta que se acerca más a su situación.*

Debido a la EM, durante las últimas 4 semanas, usted...

1. ¿Ha tenido dificultades para caminar?

2. ¿Ha tenido dificultades en las actividades fuera de casa tales como ir de compras o ir a ver una película?

3. ¿Ha tenido dificultades para desplazarse en su casa?

4. ¿Ha padecido problemas de equilibrio o al caminar?

5. ¿Ha tenido dificultades con las actividades dentro de casa, tales como hacer manualidades, atender las plantas o el jardín?

6. ¿Ha tenido dificultades con las actividades laborales: por ejemplo, integración, interrupción o limitación?

7. ¿Se ha sentido cansado con rapidez?

8. ¿Se ha sentido falto de energía?

9. ¿Se ha sentido ansioso/a?

10. ¿Se ha sentido deprimido/a o melancólico/a?

11. ¿Ha sentido ganas de llorar?

12. ¿Se ha sentido nervioso/a o irritado/a por ciertas cosas o situaciones?

13. ¿Ha tenido problemas de pérdida de memoria?

14. ¿Ha tenido dificultades de concentración: por ejemplo, al leer, ver una película, siguiendo una discusión?

15. ¿Ha tenido molestias en la vista: empeoramiento de visión o molestias?

16. ¿Ha tenido sensaciones desagradables: por ejemplo, calor, frío?

17. ¿Ha hablado con sus amigos/as?

18. ¿Se ha sentido comprendido/a por sus amigos/as?

19. ¿Se ha sentido apoyado/a por sus amigos/as?

20. ¿Ha hablado con su esposo/a, compañero/a o su familia?

21. ¿Se ha sentido comprendido/a por su esposo/a, compañero/a o su familia?

22. ¿Se ha sentido apoyado/a por su esposo/a, compañero/a o su familia?

23. ¿Se ha sentido satisfecho/a con su vida sentimental?

24. ¿Se ha sentido satisfecho/a con su vida sexual?

25. ¿Ha sentido que su situación es injusta?

26. ¿Se ha sentido amargado/a?

27. ¿Se ha sentido molesto/a por las miradas de otras personas?

28. ¿Se ha sentido avergonzado en público?

29. ¿Está usted satisfecho/a con la información acerca de su enfermedad o con el tratamiento dado por el equipo médico (doctores, enfermeras, psicólogos) en el cuidado de su EM?

30. ¿Se ha sentido comprendido/a por el personal sanitario (doctores, enfermeras, psicólogos) en el cuidado de su EM?

31. ¿Se ha sentido satisfecho con los tratamientos que recibe?
